# Weekly Cladribine Followed by Rituximab for the Treatment of Hairy Cell Leukemia

**DOI:** 10.1002/jha2.70311

**Published:** 2026-05-28

**Authors:** Jacqueline A. Turner, Jennifer Santos, Vincenzo Pizzuti, Emily Paton, William A. Robinson

**Affiliations:** ^1^ Division of Medical Oncology Department of Medicine University of Colorado School of Medicine Anschutz Medical Campus Aurora Colorado USA; ^2^ Internal Medicine Residency Training Program Department of Medicine Mayo Clinic College of Medicine and Science Rochester Minnesota USA; ^3^ Internal Medicine Residency Training Program Department of Medicine University of Colorado School of Medicine Colorado USA; ^4^ Division of Medical Oncology Department of Medicine Vanderbilt University Medical Center Nashville Tennessee USA; ^5^ Internal Medicine Residency Training Program Department of Medicine Oregon Health Sciences University Portland Oregon USA

**Keywords:** BRAF, cladribine, hairy cell leukemia

## Abstract

**Introduction:**

Hairy cell leukemia (HCL) is an uncommon hematopoietic stem cell disease known to have an underlying somatic *BRAF^V600E^* driver mutation. Currently, the most accepted treatment is five or seven consecutive days of outpatient infusion of cladribine (e.g., continuous infusion), despite frequent antibiotic use and hospitalization with this regimen. As a result, a few investigators have adopted weekly infusion of cladribine with similar or improved results and fewer side effects.

**Methods:**

We conducted a retrospective, single‐institution cohort study of 36 treatment‐naïve patients with HCL treated at the University of Colorado Hospital. Eighteen patients received intermittent weekly cladribine (intermittent cladribine [IC]) for 5–7 doses and 18 received standard daily cladribine (continuous cladribine [CC]) over 5–7 days. We report clinical cohort characteristics, response rates, progression‐free survival, overall survival, toxicity, hospitalization rates, and measurable residual disease monitoring in select patients using peripheral blood quantitative *BRAF^V600E^* PCR.

**Results:**

We report here our experience with 18 patients with newly diagnosed *BRAF*‐mutated HCL treated with weekly cladribine at a single institution compared to 18 HCL patients who received continuous infusions. Baseline hematologic parameters and overall survival were comparable between groups (*p* = 0.1135), but there was a higher rate of complete remission for patients treated with IC (94.4% vs. 61.1%) with significantly improved progression‐free survival (*p* < 0.0001). We observed comparable rates of neutropenia, neutropenic fever, antibiotic use, and G‐CSF administration. There were fewer hospitalizations for patients treated with IC (4/18) compared to patients treated with CC (6/18). Rituximab exposure differed between groups, with 72% of patients in the IC cohort receiving rituximab compared with 27.2% in the CC cohort (although treatment status was documented for only 66.7% of patients in the CC cohort). Peripheral blood *BRAF^V600E^* allele burden significantly declined and correlated with clinical remission, but this was only performed in select patients who were treated in recent years.

**Conclusions:**

In this retrospective analysis, IC dosed weekly was associated with improved progression‐free survival and higher rates of complete remission compared to CC with comparable toxicity and fewer hospitalizations. These findings suggest a potential clinical benefit from this regimen. However, interpretation of these findings is limited by the non‐randomized design and differences in rituximab exposure between groups, which may have contributed to the observed outcomes and limited definitive conclusions.

Trial Registration: The authors have confirmed clinical trial registration is not needed for this submission

1

Hairy cell leukemia (HCL) is an uncommon hematopoietic stem cell disease known to have an underlying somatic *BRAF^V600E^* driver mutation. A recent study [1] identifies continuous infusion (e.g., outpatient consecutive daily infusions) of cladribine for 5 or 7 days as the most accepted first‐line of treatment, despite significant infectious complications with this regimen. We adopted weekly outpatient cladribine infusion with similar or improved results and fewer side effects. We report 18 patients with newly diagnosed *BRAF*‐mutated HCL treated with weekly cladribine have significantly improved progression‐free survival (PFS) compared to 18 patients treated with continuous infusions (daily intravenous administration over 5–7 days).

HCL was first described in 1923 and has since undergone significant advances in understanding [2, 3]. HCL is a male‐predominate lymphoproliferative hematopoietic stem cell disorder with a high frequency of somatic mutation with *BRAF^V600E^* [2, 3, 4, 5, 6, 7]. Treatment has evolved from splenectomy to interferon and pentostatin to cladribine and, more recently, combinations of the latter with rituximab [2, 3, 8]. Limited series have also shown excellent results with BRAF inhibitors [2, 3, 4, 5, 6]. The most accepted and widely used treatment over the past 20 years has been continuous infusion of cladribine over 5–7 days, which results in high remission rates [3] though it is complicated by considerable toxicity [2, 3, 9, 10]. More recently, the addition of rituximab has increased the remission rate and survival but has not decreased toxicity. Following several HCL patient deaths from continuous cladribine (CC) infusion 15 years ago, several investigators hypothesized that weekly cladribine infusions would lead to similar response rates with less toxicity. Previous studies comparing the outcomes and toxicity between continuous versus weekly cladribine infusion have shown mixed results [3, 9, 10, 11, 12, 13]. We report here our experience with newly diagnosed HCL treated with weekly cladribine (intermittent cladribine [IC]) or daily intravenous cladribine over 5–7 days (CC) at a single institution over the past 23 years.

Thirty‐six patients with HCL were retrospectively identified at the University of Colorado Hospital and were treated at the institute. Retrospective data analysis was conducted under waived consent and/or authorization with secondary research intake (#26‐0219). Eighteen patients were treated with IC dosed weekly for 5–7 infusions of cladribine, or were treated with CC infusion completed at daily outpatient infusions over 5–7 days. The median age at diagnosis for IC was 58 (range 32–84, average 57.2) with 83.3% being male patient and 72.2% had also received rituximab at a later point in their treatment course. The median age at diagnosis for CC was 45 (range 32–66, average 45.2) with 83.3% male patients, and 27.2% (33.3% unknown) had received rituximab. Of the total cohort (*n* = 36), 94.4% were Caucasian, with one Hispanic patient and one Asian patient included in the CC treatment group. *BRAF^V600E^* mutations were frequent within the cohort (IC % mutated/wild‐type/unknown: 66.6/11.1/22.2; CC % mutated/wild‐type/unknown: 50/0/50). The white blood cell count (WBC), hemoglobin (Hgb), and platelet counts at the time of diagnosis were comparable between IC and CC groups. Overall, average WBC/Hgb/platelet counts for IC were 4.6/12.2/68.7, and CC were 3.8/10.8/79.3; notably, there were three patients for whom values were unknown at the time of diagnosis in the CC group (Figure 1A–C). However, there were no statistically significant differences between the groups. All patients were treatment naïve prior to cladribine therapy.

**FIGURE 1:**
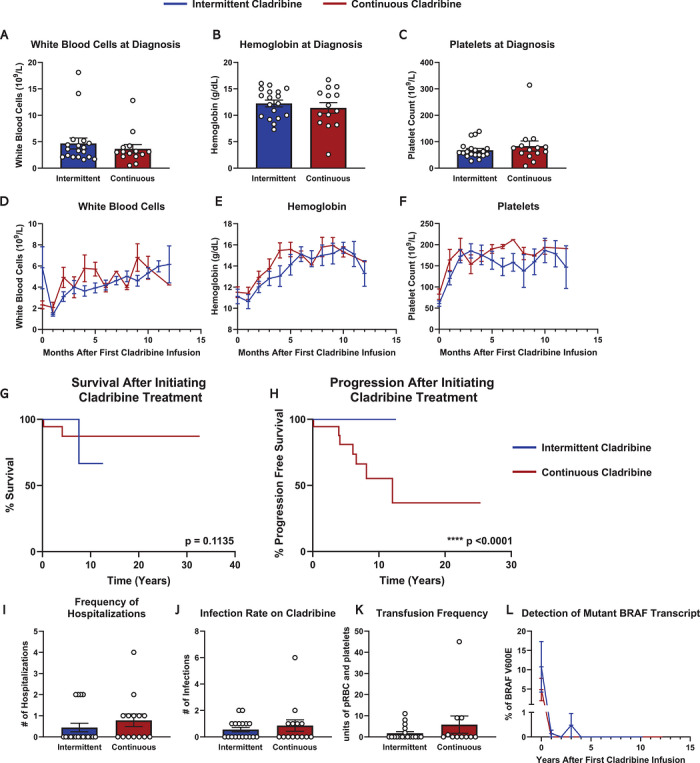
Intermittent cladribine versus continuous cladribine in the treatment of hairy cell leukemia. (A–C) Laboratory values at the time of diagnosis showing (A) white blood cell count, (B) hemoglobin, and (C) platelets, where blue depicts patients treated with intermittent cladribine (blue) as compared to continuous cladribine (red). Statistical significance was assessed and determined to be not significant with *p* > 0.05 based on the Student's unpaired *t*‐test for panels A–C. (D–F) Longitudinal laboratory values across 12 months after initiating cladribine infusions showing (D) white blood cell counts, (B) hemoglobin, and (C) platelets between intermittent cladribine (blue) and continuous cladribine (red). Statistical significance was assessed and determined to be not significant with *p* > 0.05 based on the Student's unpaired *t*‐test for panels D–F. (G, H) Kaplan–Meier curves representing (G) overall survival and (H) progression‐free survival after first cladribine infusions for patients treated with intermittent cladribine (blue) or continuous cladribine (red), with *p*‐values representing statistical values determined by log‐rank test showing *p* = 0.8642 and *p* < 0.0001. (I–K) Quantified adverse events with cladribine infusions and measured by (J) number of hospitalizations, (K) number of infections, and (L) units of transfused blood products for patients treated with intermittent cladribine (blue) or continuous cladribine (red). (L) The percentage of BRAFV600E detected in the peripheral blood. The graph represents the averaged values across patients treated with either intermittent cladribine (blue) or continuous cladribine (red). Statistical significance was assessed and determined to be not significant with *p* > 0.05 based on the Student's unpaired *t*‐test for panels I–K.

The remission rate in patients treated with IC was 94%, whereas CC achieved a 61.1% complete response rate with 38.9% of patients experiencing progressive disease (Table 1). The WBC, Hgb, and platelet counts were comparable and not statistically significant between the two groups (Figure 1D–F and Figure S1). There was comparable overall survival between the two groups; however, there was a significant advantage to PFS after initiating cladribine (Figure 1G, H with *p* = 0.1135 and *p* < 0.0001, respectively). As such, 100% of IC patients have not progressed, versus 38.9% of CC patients who have progressive disease. The remission rates were determined by (1) laboratory‐based blood counts and (2) serial measurement of mutated *BRAF* allelic frequency in peripheral blood samples (Figure 1I). Importantly, most IC patients received rituximab for 4 weeks after initial treatment with cladribine. In total, 4/18 IC patients required hospitalization during treatment induction, whereas 6/18 CC patients required hospitalization (Figure 1J). Neutropenia was observed in 88.9% of IC patients with neutropenic fever in 44.4% of patients. Neutropenia was identified in 92.9% of CC patients with recorded data (13/14 and 4 patients with unknown lab values), and neutropenic fever was observed in 42.9% (6/14 patients). Infection rate was comparable between groups (Figure 1K), where eight IC and six CC patients experienced infection requiring antibiotic therapy. In total, in both IC and CC groups, 50% of patients were treated with antibiotics (9/18 and 6/14 patients, respectively). G‐CSF agents were administered to 38.9% (7/18) of IC patients and 38.5% of CC patients (4/13 and 5 patients with unknown documentation). There were 27.7% of IC patients that required blood product transfusions, and 36.4% of CC patients with recorded data (4/11 and 7 patients with unknown data) that required transfusion (Figure 1L).

**TABLE 1:** Patient characteristics and results (*n* = 36).

	Intermittent cladribine (18)	Continuous cladribine (18)
Age at diagnosis	57.2(32–84)	45.2(32–66)
Male	83.3(15)	83.3(15)
Treatment response		
Complete response	94.4(17)	61.1(11)
Progressive disease	0(0)	38.9(7)
Ongoing	5.6(1)	0(0)
Adverse events and side effects		
Neutropenia	16/18 (88.9)	13/14 (92.9)
Fever	8/18 (44.4)	6/14 (42.9)
Antibiotics	9/18 (50)	7/14 (50)
Cladribine reaction	4/18 (22.2)	2/14 (14.2)
G‐CSF agents	7/18 (38.9)	5/13 (38.5)

Peripheral blood was obtained in a subset of patients to measure mutant heterozygous *BRAF^V600E^* allele by quantitative PCR (qPCR), which has been proposed as a proxy for measurable residual disease (MRD) detection in HCL [11, 14]. At 5 years post‐cladribine infusion, all patients tested in both IC and CC groups had undetectably low levels of mutant alleles, that is no MRD in peripheral blood (Figure 1I and Figure S2). In most instances, remission was determined by return of peripheral blood counts to normal or near normal range, and *BRAF* mutant allele frequency was incorporated more recently as an added indicator of disease status in our cohort. There is a discrepancy in the number of patients in each group, as this assay was infrequently performed for patients routinely treated with CC. Regardless, patients treated in either group had significant reduction in the percentage of mutant alleles detected in peripheral blood samples, which correlated well with clinical responses. This observation further supports the use of serial *BRAF* mutant allele frequency testing routinely in HCL patients and may serve as a less invasive MRD monitoring tool that obviates the need for repeat bone marrow biopsies.

In addition to our 36‐patient cohort, we identified three patients who had progressed after treatment with CC and were rechallenged with IC. The median age at diagnosis for IC was 53 (range 38–54, average 48.3), and all were male patients. These patients achieved complete responses upon IC rechallenge with PFS periods of 10.1 years, 5.7 years, and 0.08 years (Figure S3A–C). Overall, 2/3 CC patients required hospitalization, while on IC, there were no hospitalization events. In addition, 2/3 patients experienced infection with neutropenic fever while on CC but not on IC.

The treatment of HCL is a success story, with improved responses with interferon, chemotherapy, and now targeted therapy. As most HCL patients are older with comorbidities, therapy should be both effective and low‐risk for both short‐term and long‐term complications. Our data suggest that weekly cladribine followed by rituximab meets these criteria, often avoiding hospitalization and continuous transfusion or antibiotic support. Prior comparisons of continuous versus weekly cladribine yielded mixed results, with limited studies over the past decade. Two prospective randomized trials showed no significant differences between the two regimens [11, 12], while Zinzani et al. and Lauria et al. reported fewer neutropenic and infectious complications with weekly dosing [9, 10, 12, 13, 15]. Our findings align with these trends and further demonstrate longer PFS with weekly cladribine group, albeit with no difference in overall survival. Given the effectiveness and safety of weekly cladribine infusion, the weekly schedule represents a feasible strategy supported by institutional experience but warrants further studies. Although prospective randomized validation would be ideal, conducting such trials may be challenging given the rarity of HCL; nevertheless, further prospective investigation would be valuable to confirm these findings. We will continue to expand and update our series. Early data also suggest that peripheral blood *BRAF* mutation levels may serve as a non‐invasive marker of response and warrant further study.

## Author Contributions

**Jacqueline A. Turner**: conceptualization, methodology, investigation, formal analysis, visualization, writing – original draft, writing – review and editing. **Jennifer Santos**: conceptualization, methodology, investigation, formal analysis, writing – original draft, writing – review and editing. **Vincenzo Pizzuti**: conceptualization, methodology, investigation, formal analysis, writing – original draft, writing – review and editing. **Emily Paton**: conceptualization, methodology, investigation, formal analysis, writing – original draft, writing – review and editing. **William A. Robinson**: conceptualization, methodology, writing – review and editing.

## Funding

This study was funded by the Amy Davis Foundation, the Moore Family Foundation, and the Heidi Horner Foundation to William A. Robinson. Additional funding was provided in part to Jacqueline A. Turnerby the Hertz Graduate Fellowship.

## Ethics Statement

This study was reviewed and approved for waived consent and/or authorization with secondary research intake by the University of Colorado Institutional Review Board (#26‐0219). This study was conducted in accordance with the ethical principles of the Declaration of Helsinki.

## Conflicts of Interest

Jacqueline A. Turner and William A. Robinson report consulting fees from Blood Cell Therapeutics. The other authors declare no conflicts of interest.

## Supporting information




**Supporting File 1**: jha270311‐sup‐0001‐figuresS1‐S3.pdf

## Data Availability

The data presented in this study are available both within the article and in the Supporting Information. Source data are provided.
