# Supplementary material for: Weekly Cladribine Followed by Rituximab for the Treatment of Hairy Cell Leukemia
Source: EJHaem. 2026 May 28;7(3):e70311. doi: 10.1002/jha2.70311 (PMC13238836; doi:10.1002/jha2.70311)
Supplement: Supplementary file 1 — Supporting File 1: jha270311‐sup‐0001‐figuresS1‐S3.pdf [file JHA2-7-e70311-s001.pdf]

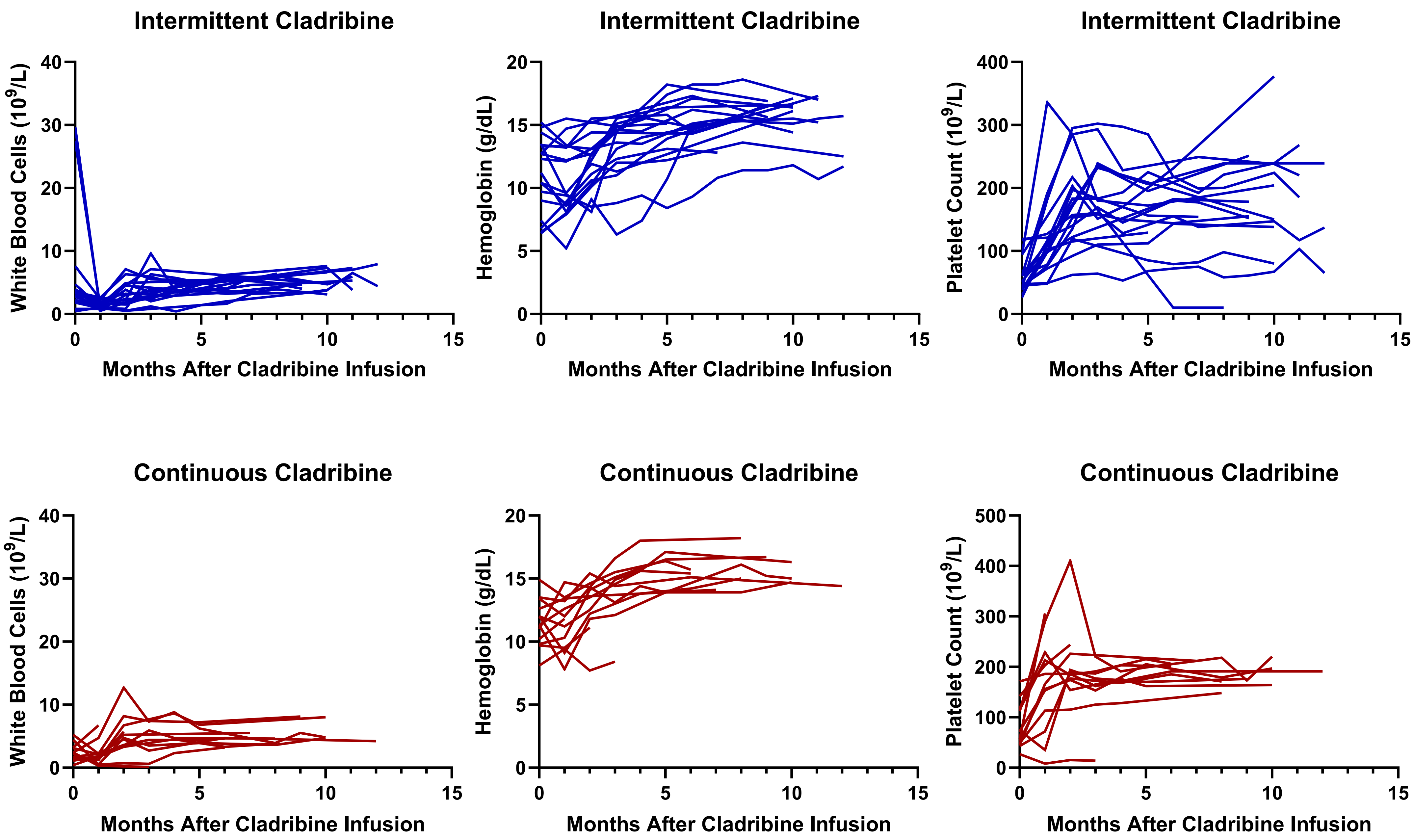

Supplemental Figure 1. Longitudinal laboratory values with each line representing an individual patient. Data are collected across 12 months after initiating cladribine infusions that are treated either with intermittent cladribine (blue) or continuous cladribine infusion (red). Panels represent white blood cells (left panels), hemoglobin (middle panels), or platelets (right panels).

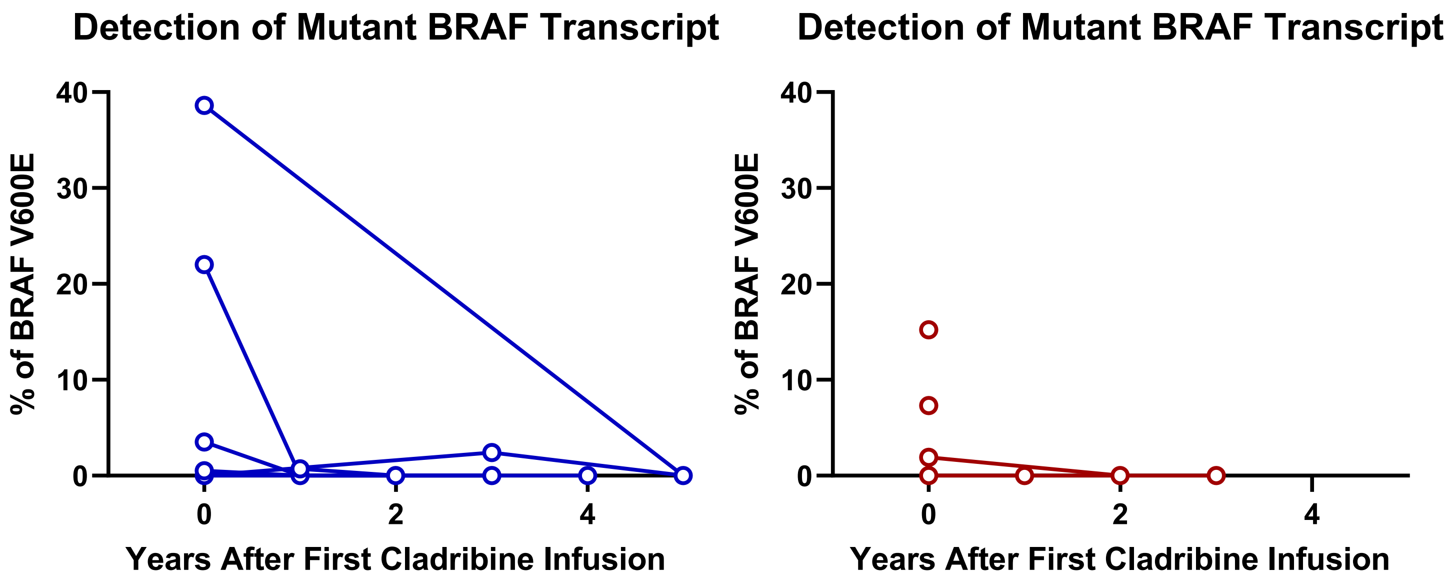

Supplemental Figure 2. Percent of *BRAF*^{V600E} detected in the peripheral blood with each line representing an individual patient. Data are collected across 4 months after initiating cladribine infusions that are treated either with intermittent cladribine (blue) or continuous cladribine infusion (red).

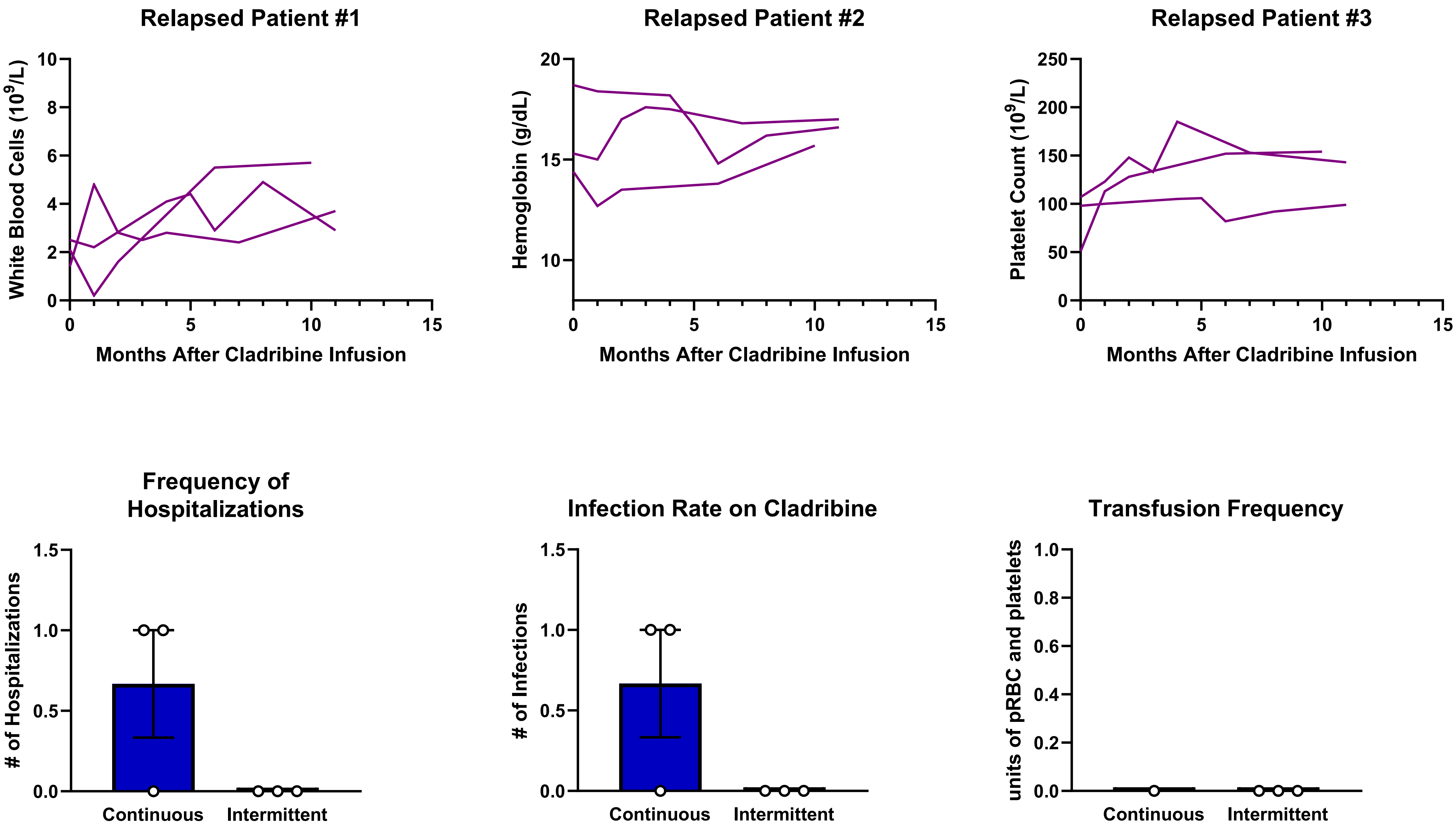

Supplemental Figure 3. Relapsed patients re-challenged with intermittent cladribine.
